# Identifying multiple collagen gene family members as potential gastric cancer biomarkers using integrated bioinformatics analysis

**DOI:** 10.7717/peerj.9123

**Published:** 2020-05-25

**Authors:** Zhaoxing Li, Zhao Liu, Zhiting Shao, Chuang Li, Yong Li, Qingwei Liu, Yifei Zhang, Bibo Tan, Yu Liu

**Affiliations:** 1 Department of General Surgery, The Fourth Affiliated Hospital of Hebei Medical University, Shijiazhuang, China; 2 Key Laboratory of Carcinogenesis and Translational Research (Ministry of Education), Department of Gastrointestinal Surgery, Peking University Cancer Hospital and Institute, Beijing, China; 3 Key Laboratory of Carcinogenesis and Translational Research (Ministry of Education), Department of Renal Cancer and Melanoma, Peking University Cancer Hospital and Institute, Beijing, China; 4 The Second Hospital of Shijiazhuang, Shijiazhuang, China; 5 Hebei General Hospital, Shijiazhuang, China

**Keywords:** Gastric cancer, Bioinformatics, Survival, Biomarker

## Abstract

**Background:**

Gastric cancer is one of the most common malignant cancers worldwide. Despite substantial developments in therapeutic strategies, the five-year survival rate remains low. Therefore, novel biomarkers and therapeutic targets involved in the progression of gastric tumors need to be identified.

**Methods:**

We obtained the mRNA microarray datasets GSE65801, GSE54129 and GSE79973 from the Gene Expression Omnibus database to acquire differentially expressed genes (DEGs). We used the Database for Annotation, Visualization, and Integrated Discovery (DAVID) to analyze DEG pathways and functions, and the Search Tool for the Retrieval of Interacting Genes (STRING) and Cytoscape to obtain the protein–protein interaction (PPI) network. Next, we validated the hub gene expression levels using the Oncomine database and Gene Expression Profiling Interactive Analysis (GEPIA), and conducted stage expression and survival analysis.

**Results:**

From the three microarray datasets, we identified nine major hub genes: COL1A1, COL1A2, COL3A1, COL5A2, COL4A1, FN1, COL5A1, COL4A2, and COL6A3.

**Conclusion:**

Our study identified COL1A1 and COL1A2 as potential gastric cancer prognostic biomarkers.

## Introduction

Gastric cancer (GC) is the fifth most common malignant cancer and the third leading cause of cancer-related mortality worldwide (Bray et al., 2018). In 2018, there were more than 1,000,000 new cases of GC and approximately 783,000 deaths (Bray et al., 2018; Siegel, Miller & Jemal, 2015). GC poses a great threat to public health, particularly in East Asia where the incidence has increased remarkably. Over the last decade, considerable progress has been made with finding and applying GC biomarkers in clinical diagnosis and treatment. For example, HER2, a member of the human EGFR family, was recognized as the most significant GC biomarker. GC’s HER2 overexpression rate reported across the literature fluctuates between 9% and 38% (Gravalos & Jimeno, 2008; Okines et al., 2013). Trastuzumab, a HER2-targeting drug beneficial for HER2-positive GC patients, is the only targeted drug currently approved for advanced GC treatment (Gomez-Martín et al., 2014). However, we still do not fully understand HER2’s role in gastric carcinogenesis. Programmed death ligand 1 (PD-L1) is overexpressed in approximately 40% of GC cases, designating it as a GC biomarker (Raufi & Klempner, 2015). PD-L1 and programmed cell death protein 1 (PD-1) affect immune tolerance. Tumors evade immune surveillance through the PD-1 pathway. The anti-PD-1 monoclonal antibody Pembrolizumab has shown clinical efficacy in GC patients with high PD-1 expression (Fife & Pauken, 2011). PD-1 pathway-blocking GC treatments and the potential biomarkers MET and E-cadherin (Durães et al., 2014; Ferreira et al., 2005) deserve further study. It is important to explore more clinically valuable GC biomarkers and therapeutic targets.

Microarray technology and bioinformatics analysis have recently become popular tools in cancer research and are used to identify differentially expressed genes (DEGs). These tools can also identify underlying biomarkers and therapeutic targets and their roles in biological processes, molecular functions, and different pathways.

In order to avoid potential false positives from using one single microarray, we screened three mRNA public datasets in our study to obtain DEGs between GC tissues and adjacent noncancerous tissue samples. Additionally, we carried out Gene Ontology (GO), Kyoto Encyclopedia of Genes and Genomes (KEGG), and protein–protein interaction (PPI) network analyses to show the molecular pathogenesis underlying carcinogenesis. Overall, we identified 159 DEGs and nine hub genes as potential GC biomarkers.

## Materials & Methods

### Obtaining microarray data

We downloaded three gene expression profiles (GSE65801, GSE54129, and GSE79973) from the Gene Expression Omnibus (GEO) dataset, an open data storage platform. GSE65801’s microarray dataset consisted of 32 GC tissue samples and 32 paired noncancerous tissue samples (Li et al., 2015). GSE54129’s dataset consisted of 111 GC tissues and 21 normal tissue samples. GSE79973’s gene expression profile consisted of 10 GC samples and 10 normal adjacent samples (He et al., 2016).

### Identifying DEGs

We utilized an online tool called GEO2R (https://www.ncbi.nlm.nih.gov/geo/geo2r/) to calculate the DEGs between GC tissues and normal samples (Barrett et al., 2013). If one gene had more than one probe set or if one probe set did not have the corresponding gene symbols, we averaged or removed them, respectively. We set the cut-off criteria as: —log_2_FC—>1.5 and adj. *p*-value <0.05 (fold change (FC) = GC tissue sample expression/adjacent noncancerous sample expression).

### Functional DEG annotation using KEGG and GO analyses

GO enrichment analysis and KEGG pathway enrichment analyses were conducted using the Database for Annotation, Visualization, and Integrated Discovery (DAVID, version 6.8), which provides functional annotations for DEGs (Huang et al., 2007; Kanehisa, 2002). We identified promising signaling pathways and functional annotations related to the DEGs. *P* < 0.05 was considered statistically significant.

### PPI network construction and module analysis

We used the Search Tool for the Retrieval of Interacting Genes (STRING) database to construct the PPI network, and applied Cytoscape to visualize the network (Szklarczyk et al., 2015). We set the cut-off criterion as confidence score >0.4. Next, we utilized the Molecular Complex Detection (MCODE) tool to identify the significant PPI network module with a node score cutoff = 0.2, a degree cutoff = 10, a maximum depth = 100, and a k-core = 2 (Bader & Hogue, 2003). We then used DAVID to perform the functional and pathway enrichment analyses for the significant module. We chose hub genes and constructed a co-expression network of significant genes using cBioPortal (http://www.cbioportal.org) (Cerami et al., 2012).

### Hub gene validation and analysis

We used the online database Oncomine (http://www.oncomine.org), which integrates numerous published microarray data, to validate the expression levels of the top nine GC DEGs. We then reverified the expression of the nine selected genes using Gene Expression Profiling Interactive Analysis (GEPIA, http://gepia.cancer-pku.cn), a new interactive online tool. Additionally, we continued to explore the differences in gene expression across pathological stages. Overall survival analysis of the nine hub genes was performed using the Kaplan–Meier plotter (http://kmplot.com/analysis/) (Cerami et al., 2012).

### Prediction and enrichment analysis of microRNAs related to hub genes

We used Targetscan (http://www.targetscan.org), an online database that reveals potential relationships between genes and microRNAs, to predict the microRNAs associated with hub genes. Then, we performed enrichment analysis of the predicted microRNAs using DNA Intelligent Analysis (DIANA-miRPath v3.0).

## Results

### Screening differentially expressed genes in GC

**Figure 1:**
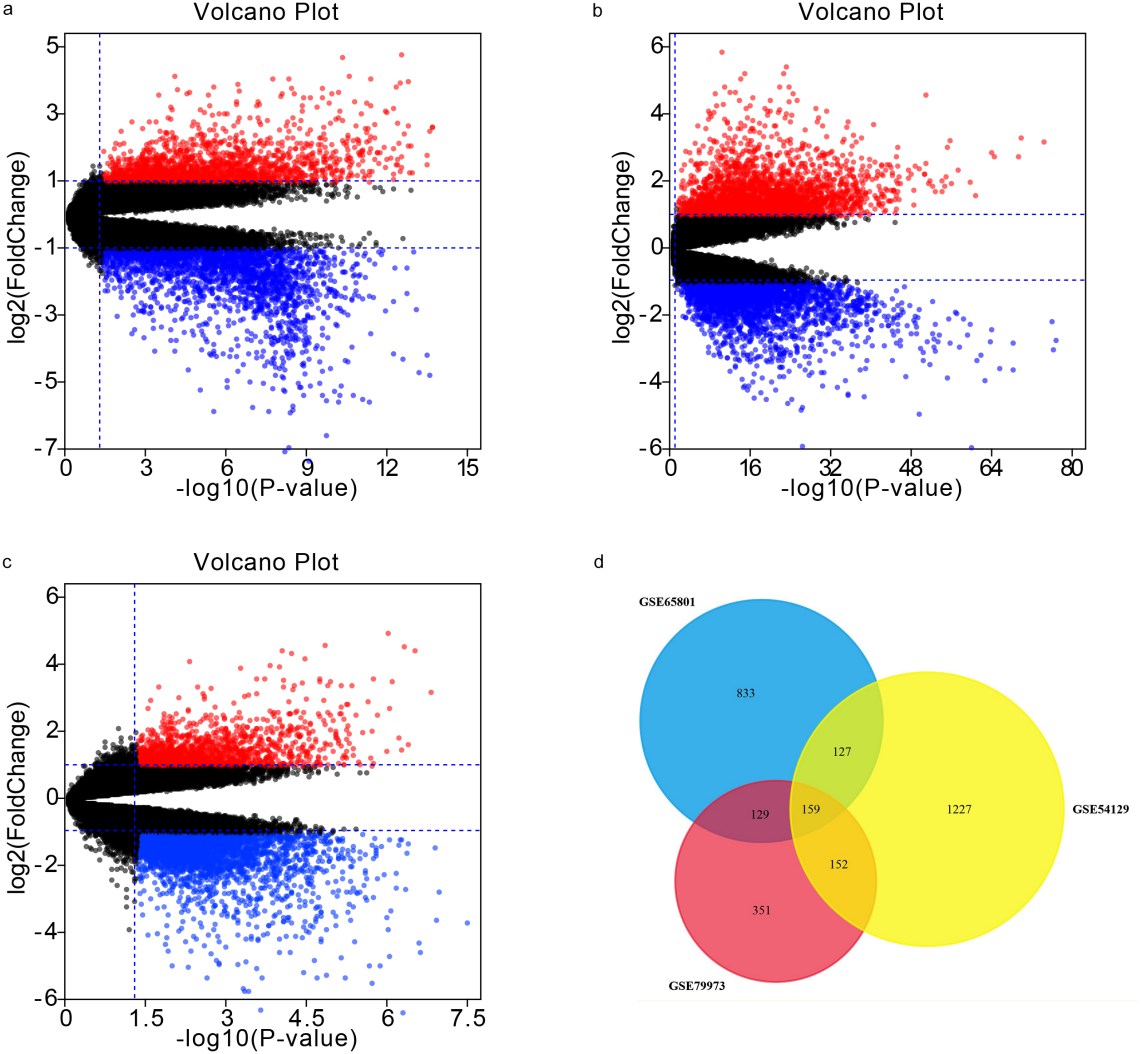
Volcano plots and Venn diagram. DEGs were selected using —log_2_FC— >1.5 and adj. *p*-value < 0.05 for the mRNA expression profiling sets GSE65801 (A) GSE54129 (C), and GSE79973 (C). The three datasets showed an overlap of 159 genes (D).

Our analyses of GSE65801, GSE54129, and GSE79973 identified 1248, 1665, and 791 DEGs, respectively. By intersecting the three GEO datasets, we also obtained 159 overlapping genes: 105 up-regulated genes and 54 down-regulated genes (Table 1). The DEGs are shown in volcano plots and a Venn diagram in Fig. 1.

**Table 1:** Screening of differentially expressed genes in gastric cancer.

DEGs	List of gene symbols
Up-regulated DEGs	COL8A1, INHBA, GREM1, COL1A1, SFRP4, SPP1, THBS2, SULF1, BGN, CTHRC1, WISP1 PRRX1, FAP, HOXC6, CRISPLD1, EDNRA, FN1, SPOCK1, ASPN, COL10A1, CST1, THY1, RARRES1, COL12A1, FNDC1, COL1A2, MFAP2, COL6A3, PDE3A, CDH11, COL4A1, OLFML2B, ADAMTS2, VCAN, TNFAIP6, IGF2BP3, TIMP1, NOX4, COL5A2, HOXC10, ADAM12, SNX10, NID2, CPXM1, CLDN1, PMEPA1, SERPINH1, COL5A1, CHN1, LOX, COL3A1, HOXA10, COMP, ANGPT2
Down-regulated DEGs	ENPP6, ALDOB, TRIM36, KCNK10, EPN3, CAPN13, LOC400043, ALDH1A1, NEDD4L, TMEM171, DGKD, PXMP2, EPB41L4B, KIAA1324, SPINK2, B3GNT6, SCNN1G, FMO5, ESRRG, ALDH6A1, LDHD, GCNT2, FBXL13, SPTSSB, MYZAP, AKR7A3, HAPLN1, THSD4, CPA2, PPP1R36, TMPRSS2, ZBTB7C, VSTM2A, LTF, CNTN3, ATP13A4, SULT1B1, STX19, HEPACAM2, RAB27B, SCNN1B, SLC26A7, CYP2C19, B4GALNT3, AKR1C1, KCNJ15, GATA5, KAZALD1, LOC643201, RDH12, XK, PIK3C2G, FER1L4, ALDH3A1, FBP2, TMED6, ITPKA, UGT2B15, AMPD1, SLC26A9, CXCL17, CA9, LIPF, PROM2, KCNE2, LYPD6B, FA2H, HHIP, GC, PSAPL1, AXDND1, RFX6, PGC, CA2, ADH7, MAL, FCGBP, PKIB, AADAC, VSIG2, ATP4A, KCNJ16, BCAS1, SULT1C2, HPGD, CYP2C18, CWH43, CAPN8, ADH1C, MUC5AC, SSTR1, ATP4B, SCIN, AKR1B10, CAPN9, VSIG1, SOSTDC1, ACER2, SLC28A2, GIF, DPCR1, HRASLS2, KRT20, GKN2, GKN1

### Functionally annotating DEGs with GO and KEGG analyses

**Figure 2:**
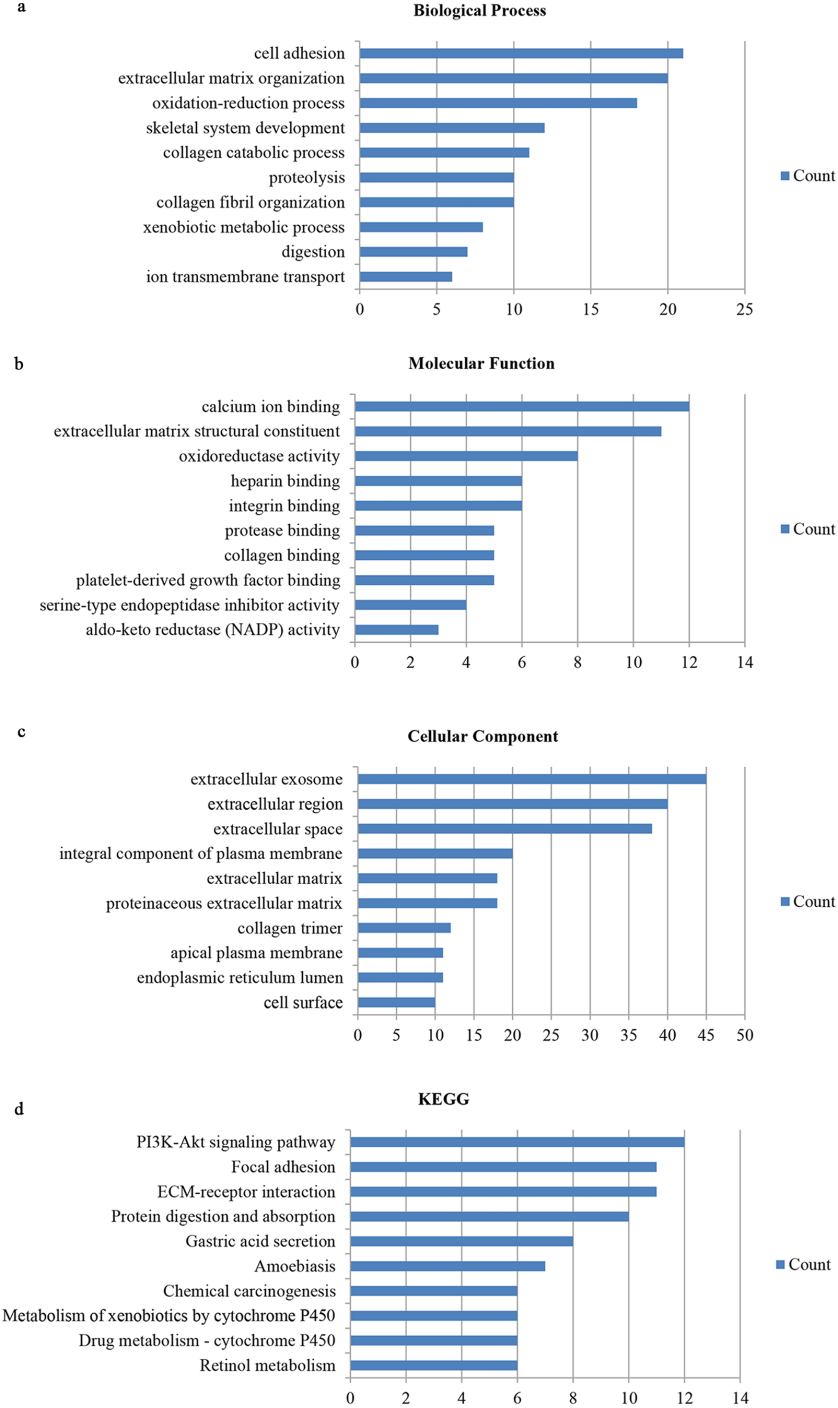
Gene ontology and DEG pathway enrichment analysis in GC. (A) Biological process. (B) Molecular function. (C) Cellular component. (D) KEGG.

We utilized the DAVID database to identify the 159 genes’ potential biological functions through GO and KEGG pathway enrichment analyses. In regards to biological processes (BP), our results showed significantly enriched variations in cell adhesion, extracellular matrix organization, oxidation–reduction processes, skeletal system development, collagen catabolic processes, proteolysis, collagen fibril organization, xenobiotic metabolic processes, digestion, and ion transmembrane transport. In terms of molecular functions (MF), our results showed close correlations with calcium ion binding, extracellular matrix structural constituents, oxidoreductase activity, heparin binding, integrin binding, protease binding, collagen binding, platelet-derived growth factor binding, serine-type endopeptidase inhibitor activity, and aldo-keto reductase (NADP) activity. Regarding cellular components (CC), the genes mainly interacted with extracellular components, such as the extracellular exosome, extracellular region, extracellular space, extracellular matrix, and proteinaceous extracellular matrix. KEGG pathway enrichment analysis showed the pathways and functions closely associated with metabolism-associated signaling, such as the PI3K-Akt signaling pathway, protein digestion and absorption, gastric acid secretion, and focal adhesion (Fig. 2).

### PPI analysis and significant module identification

**Figure 3:**
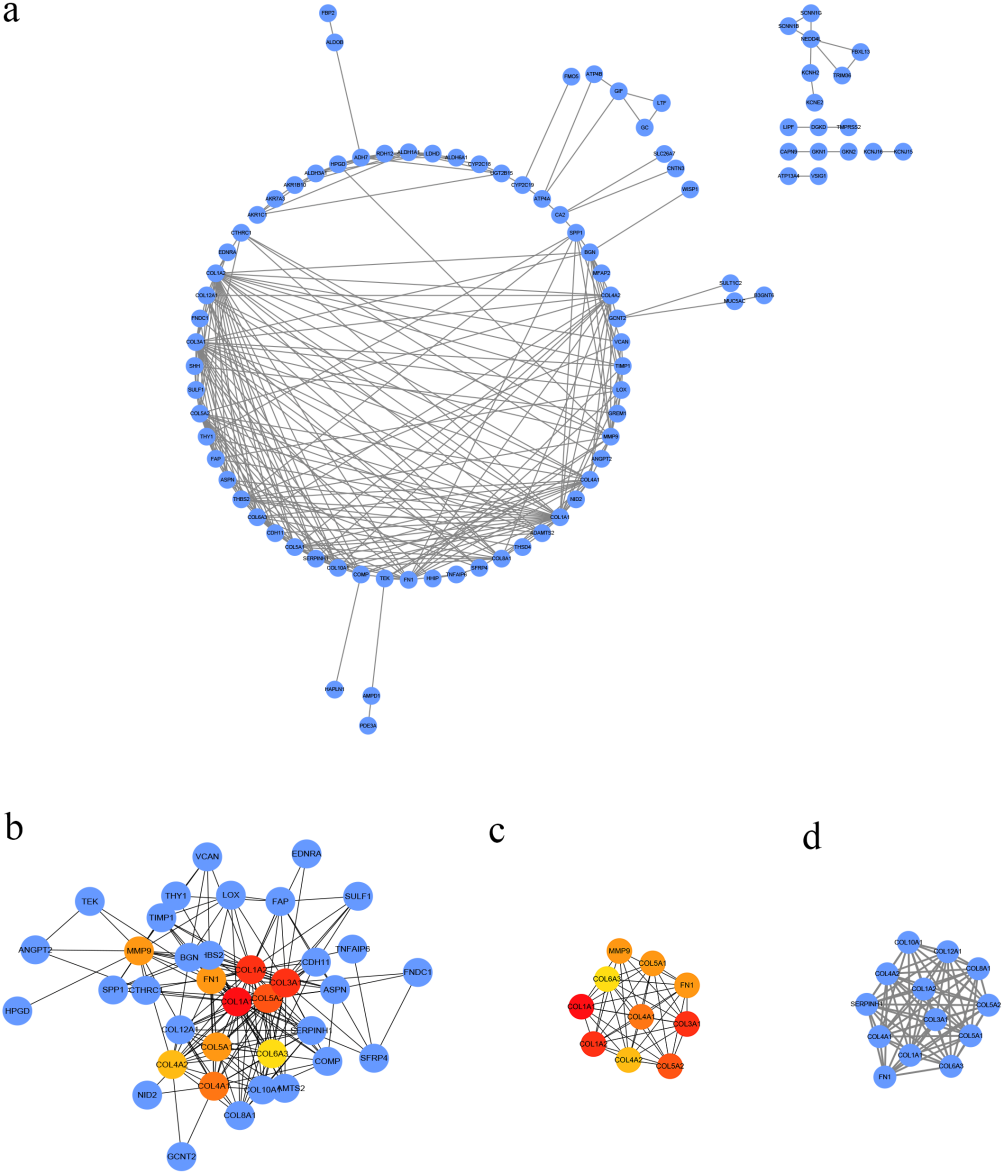
PPI network of DEGs. (A) The PPI network of DEGs constructed using Cytoscape. The PPI network included 89 nodes and 252 edges. (B) The top ten candidate hub nodes in the PPI network and their DEGs. (C) The top ten candidate hub nodes acquired in the PPI network. Red represents the highest significance, followed by tan. Yellow is the least significant. (D) The most significant module was obtained from the PPI network of DEGs using MCODE.

We conducted PPI analysis of the DEGs using the STRING database to identify the hub genes and to show their interactions in GC development. The PPI network included 89 nodes and 252 edges. We further identified the candidate hub genes by calculating the PPI network degree and set the cut-off criteria at degree ≥13. The top 10 candidate hub nodes were COL1A1, COL1A2, COL3A1, COL5A2, COL4A1, FN1, MMP9, COL5A1, COL4A2, and COL6A3. Additionally, we performed module analysis to identify the most significant module with the highest score. The identified module contained nine candidate hub genes except MMP9, indicating that it may perform critical PPI network biological functions. We suggest that the nine candidate genes are hub genes of the PPI network (Fig. 3).

**Figure 4:**
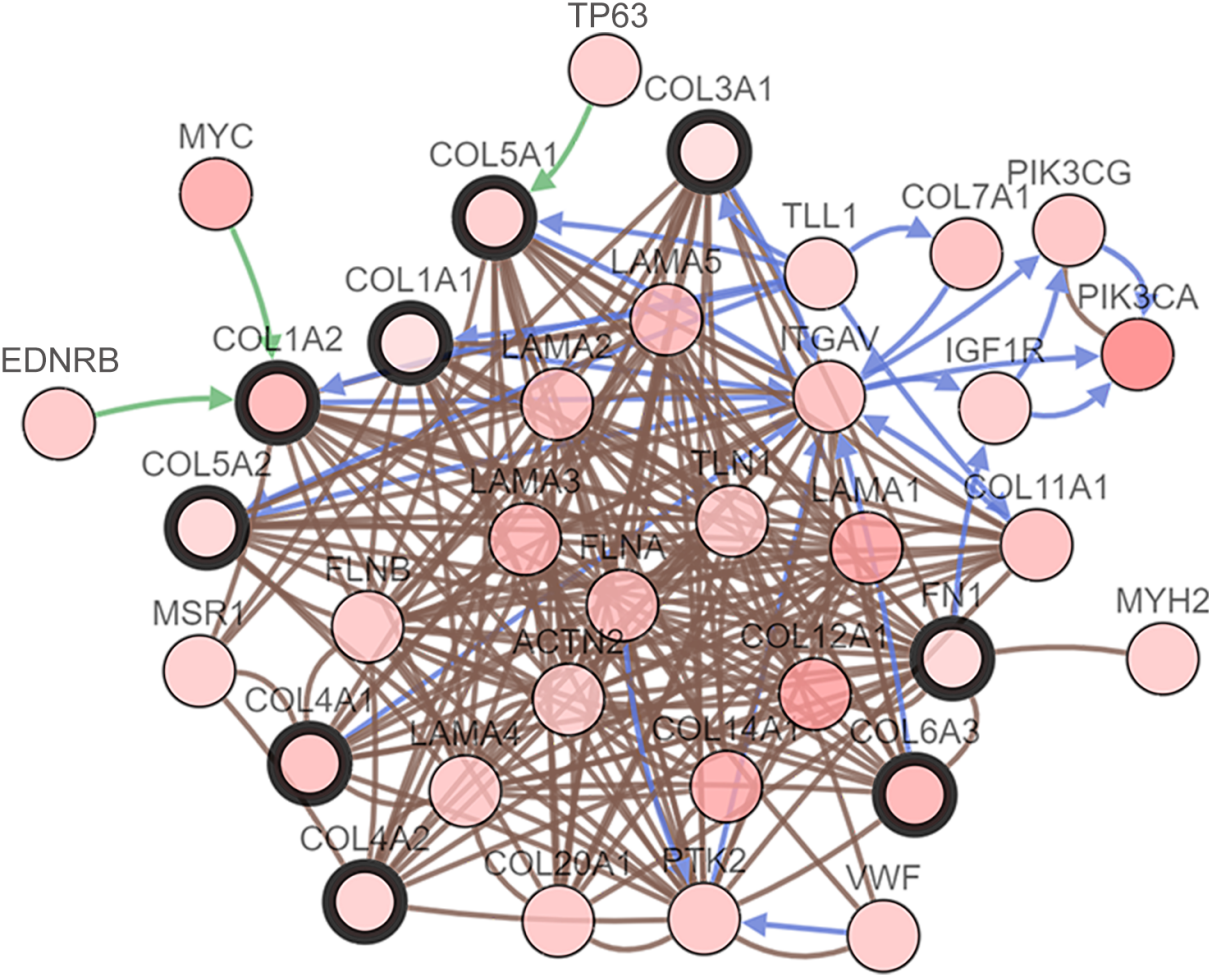
Interaction network hub gene analysis. Hub genes and their co-expression genes were analyzed using cBioPortal. Nodes with bold black outlines represent hub genes. Nodes with thin black outlines represent co-expression genes.

**Figure 5:**
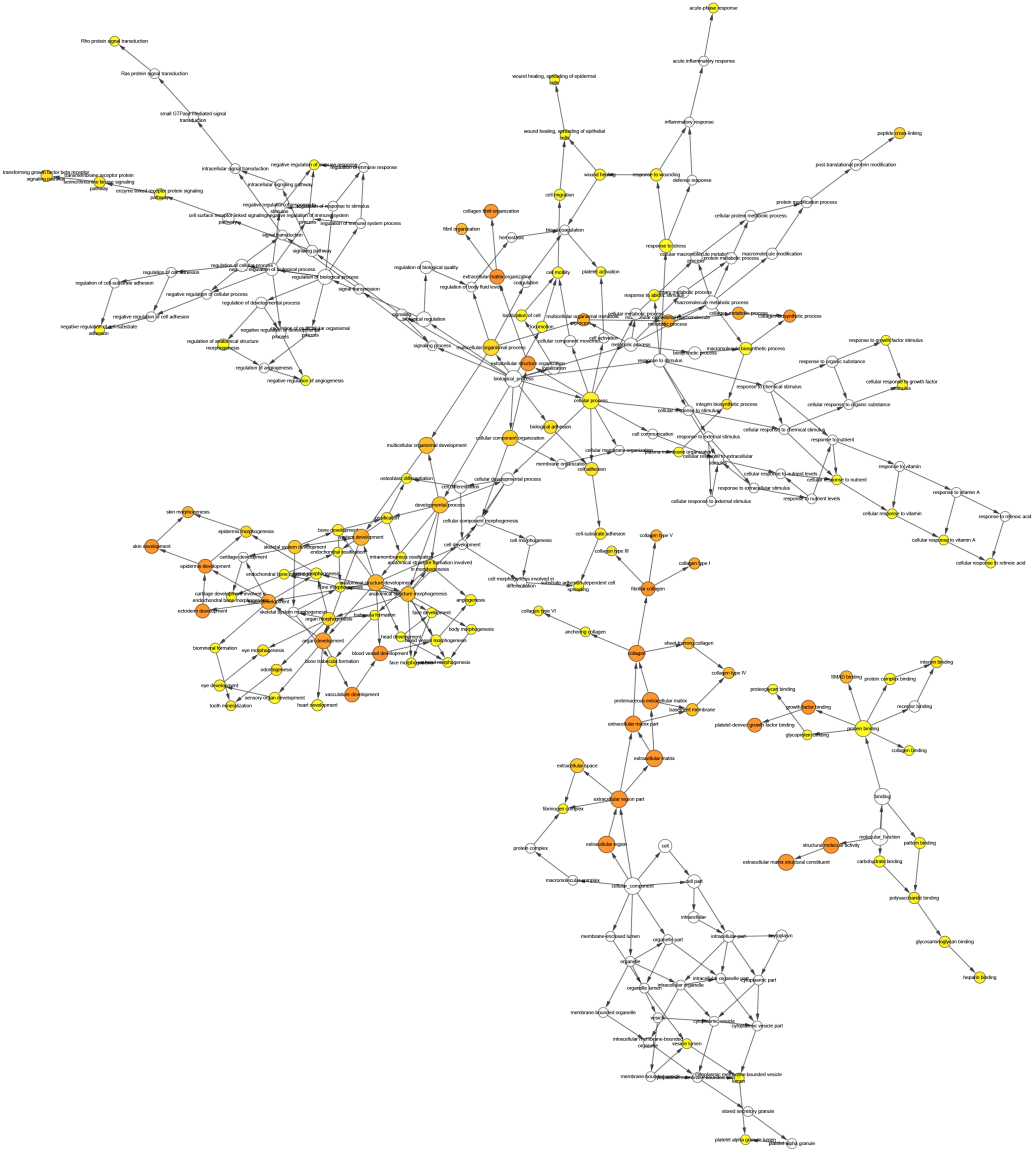
The interaction network’s biological process analysis. The node color refers to the corrected *P*-value of ontologies. *P*-value < 0.05. Orange represents the smallest *p*-value, followed by yellow, and white represents the largest *p*-value. The node size refers to the numbers of genes involved in the ontologies. The larger the node diameter, the more genes involved in the node.

We performed GO analysis on the significant module in the PPI network, and the results showed that the top module was highly involved in the collagen catabolic process, extracellular matrix organization, extracellular matrix structure, collagen fibril organization, platelet-derived growth factor binding, SMAD binding, endoplasmic reticulum lumen, extracellular matrix, and collagen trimer. KEGG pathway analysis showed that the genes in the hub module were closely connected with protein digestion and absorption, ECM-receptor interaction, and amoebiasis (Table 2). Subsequently, we utilized the cBioPortal online platform to construct the co-expression network of the nine hub genes (Fig. 4) and analyze their biological characteristics (Fig. 5).

**Table 2:** GO and KEGG pathway enrichment analysis of DEGs in the most significant module.

Category	Term	Count in gene set	*P*-value
GOTERM_BP	collagen catabolic process	11	0.000
GOTERM_BP	extracellular matrix organization	11	0.000
GOTERM_BP	collagen fibril organization	7	0.000
GOTERM_MF	extracellular matrix structural constituent	7	0.000
GOTERM_MF	platelet-derived growth factor binding	5	0.000
GOTERM_MF	SMAD binding	3	0.000
GOTERM_CC	endoplasmic reticulum lumen	12	0.000
GOTERM_CC	extracellular matrix	11	0.000
GOTERM_CC	collagen trimer	9	0.000
KEGG_PATHWAY	Protein digestion and absorption	10	0.000
KEGG_PATHWAY	ECM-receptor interaction	9	0.000
KEGG_PATHWAY	Amoebiasis	8	0.000

### Hub gene validation and analysis

**Figure 6:**
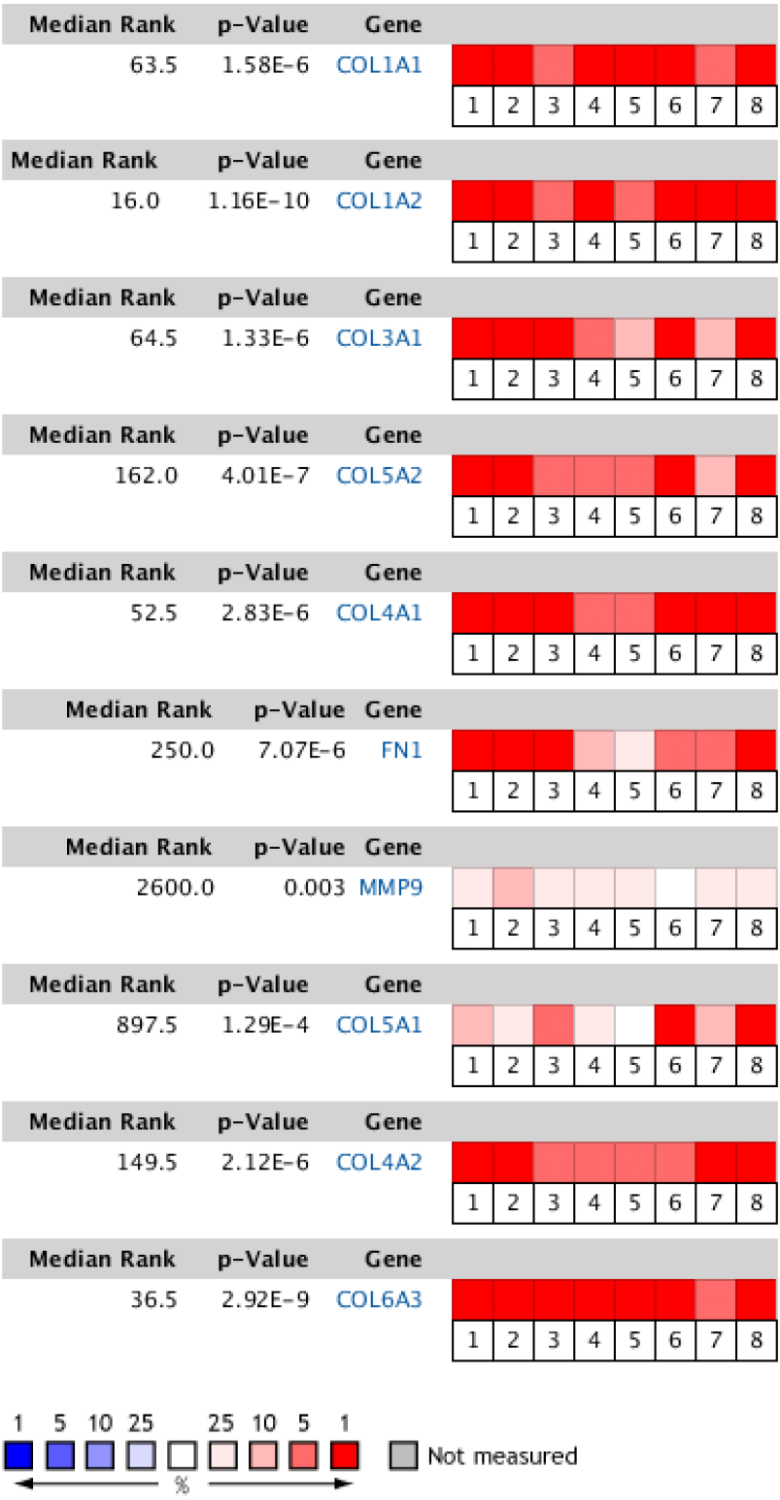
Heat map of differential expression between clinical GC samples and normal samples in the Oncomine dataset. The overexpression (red) or underexpression (blue) of target genes in eight validation datasets. In each dataset, all genes were sequenced from high to low according to their expression differences between tumor and normal tissues, and then the target gene sequencing percentiles were analyzed. Cell color was determined by the gene rank percentile for the dataset analyses (the more overexpressed the gene, the redder the dataset color, and the more underexpressed genes were blue). 1. Diffuse gastric adenocarcinoma vs. normal (Chen et al.,, 2003). 2. Gastric intestinal type adenocarcinoma vs. normal (Chen et al.,, 2003). 3. Gastric mixed adenocarcinoma vs normal (Chen et al.,, 2003). 4. Diffuse gastric adenocarcinoma vs. normal (Cho et al.,, 2011). 5. Gastric intestinal type adenocarcinoma vs. normal (Cho et al.,, 2011). 6. GC vs. normal (Cui et al.,, 2011). 7. Gastric intestinal type adenocarcinoma vs. normal ( DÉrrico et al.,, 2009). 8. GC vs. normal (Wang et al.,, 2012).

**Figure 7:**
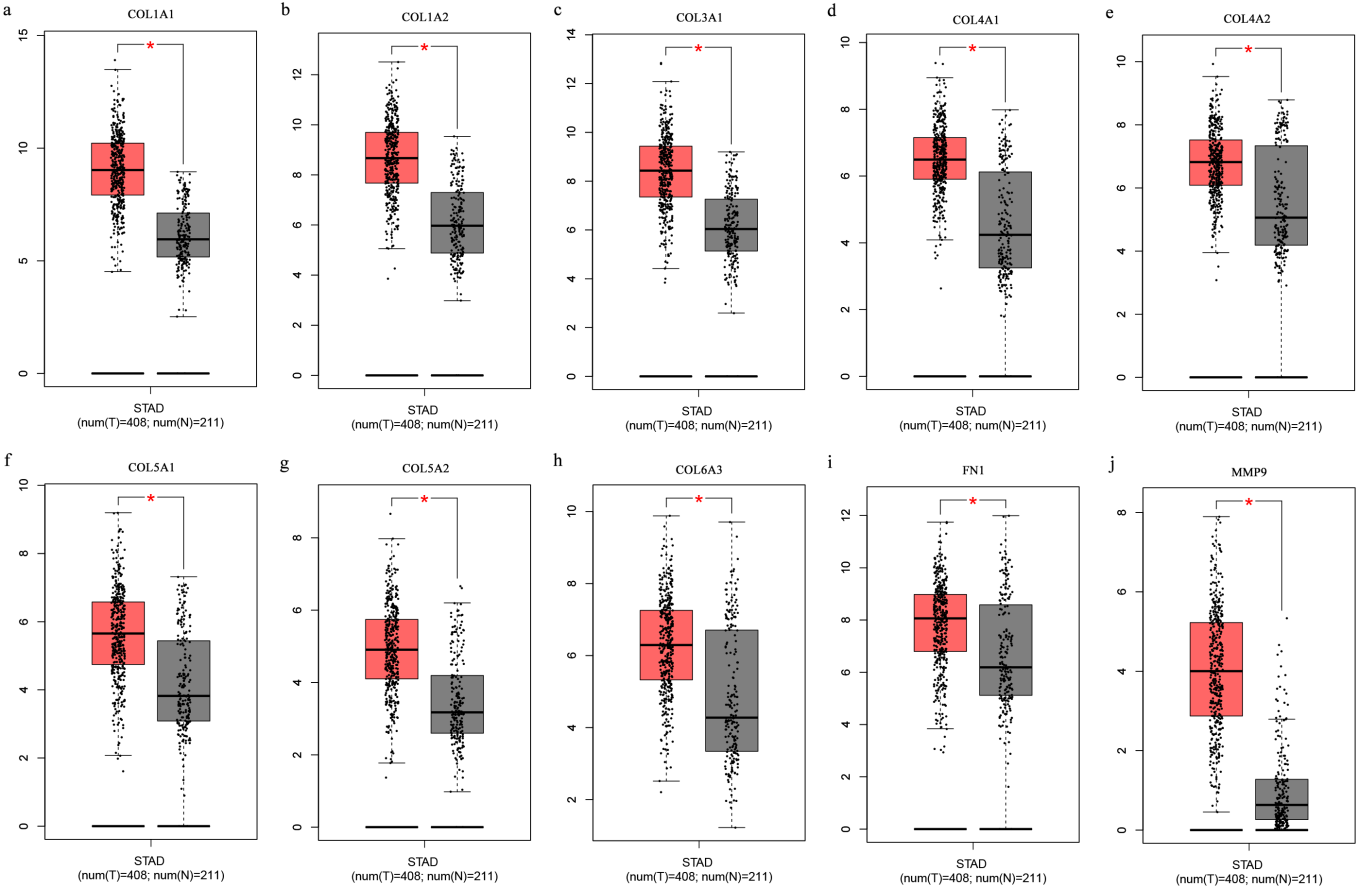
(A-J) Boxplots showing the hub gene expression differences between GC and normal tissues.

**Figure 8:**
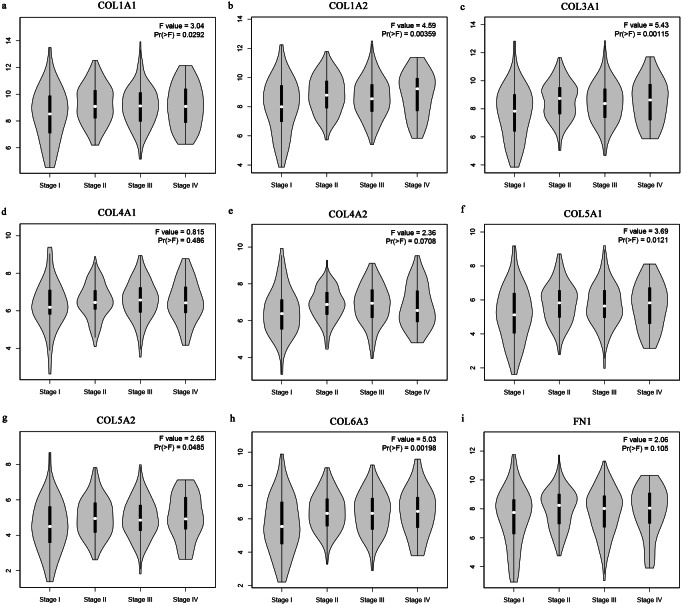
Stage plots of GC hub genes. (A) COL1A1, (B) COL1A2, (C) COL3A1, (F) COL5A1, (G) COL5A2, and (H) COL6A3 showed significant differences in different GC stages. (D) COL4A1, (I) FN1, and (E) COL4A2 were not significantly different across various stages.

**Figure 9:**
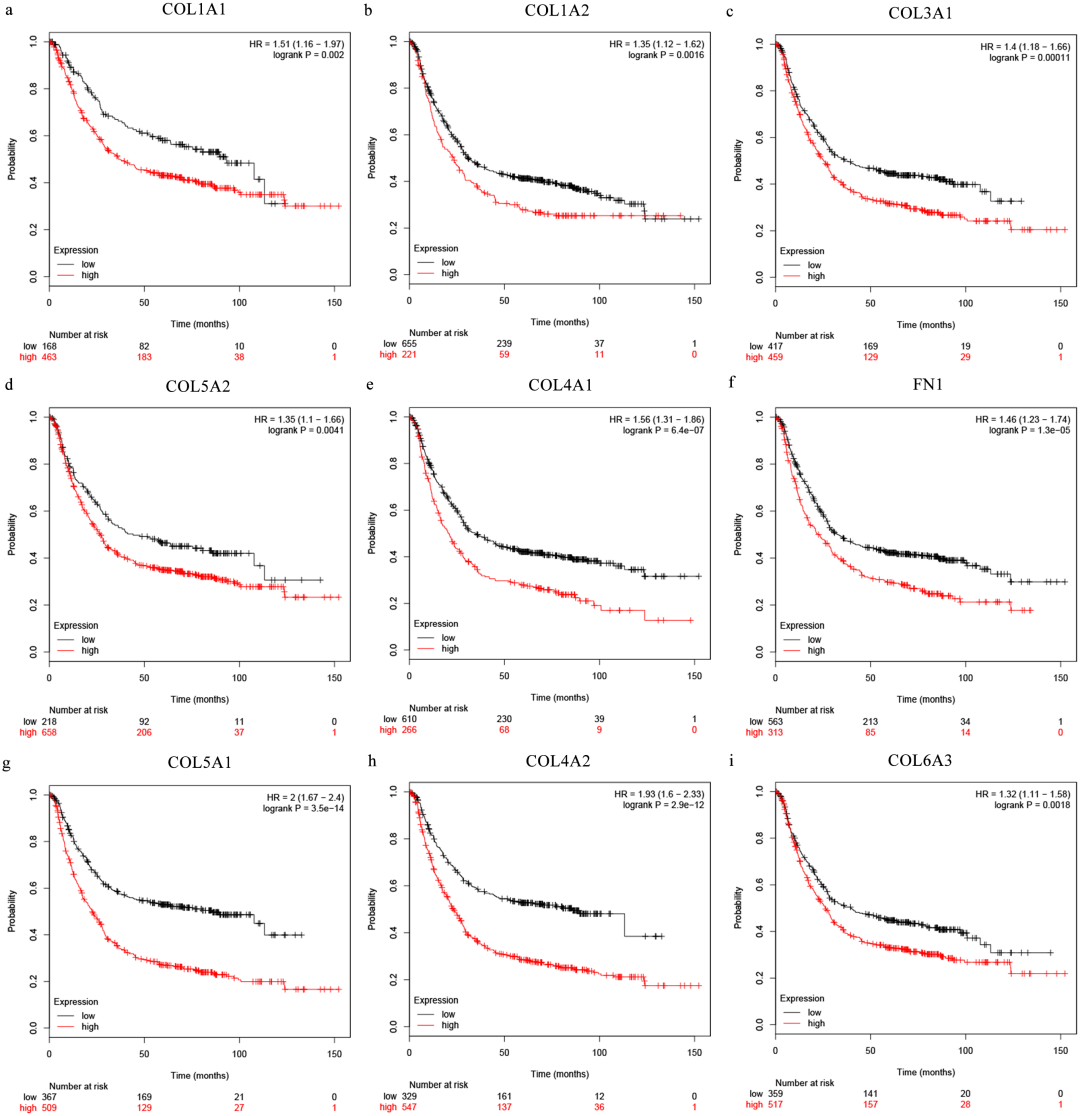
Overall survival analysis of the nine hub genes ((A) COL1A1, (B) COL1A2, (C) COL3A1, (D) COL5A2, (E) COL4A1, (F) CFN1, (G) COL5A1, (H) COL4A2, and (I) COL6A3) were plotted using the Kaplan–Meier online platform. *P* < 0.05 was considered statistically significant.

The Oncomine database collects great quantities of tumor gene expression data, while GEPIA utilizes cancer sample sequencing expression data from the Cancer Genome Atlas (TCGA) and Genotype-Tissue Expression (GTEx) projects. We selected datasets related to GC to verify their hub gene expression. Based on the Oncomine and GEPIA analysis results between cancer and normal tissue, we further verified that the hub gene expression rose significantly across the different GC datasets (Figs. 6 and 7). We also analyzed the expression levels of selected genes in different GC stages. COL1A1, COL1A2, COL3A1, COL5A1, COL5A2, and COL6A3 showed notable differences across different GC stages. COL4A1, FN1, and COL4A2 showed no clear differences across various stages (Fig. 8). We performed overall survival analysis of the nine hub genes using the Kaplan–Meier plotter and the results showed a close correlation with survival time. Figure 9 shows the remarkable difference in overall survival between the low- and high-expression groups. GC patients with high COL1A1, COL1A2, COL3A1, COL5A2, COL4A1, FN1, COL5A1, COL4A2, and COL6A3 expression levels showed worse overall survival (Fig. 9).

### Prediction and enrichment analysis of hub gene-related miRNAs

**Figure 10:**
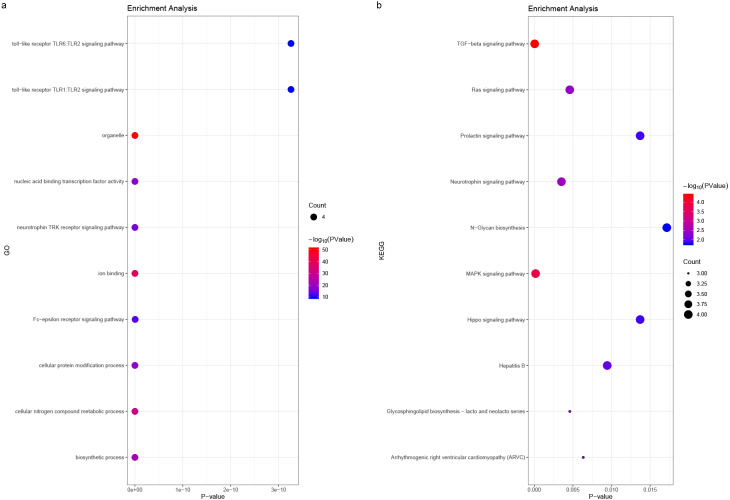
(A) miRNA GO and (B) pathway enrichment analyses closely associated with hub genes. The bubble diameter represents the number of genes involved in the enrichment term, and the bubble color represents -log10 (*p*-value).

We predicted the miRNAs associated with the hub genes’ mechanisms and regulatory network (Table 3) and conducted enrichment analysis (Fig. 10). GO analysis showed that the miRNAs were significantly enriched in the toll-like receptor TLR1:TLR2 signaling pathway, neurotrophin TRK receptor signaling pathway, and Fc-epsilon receptor signaling pathway. KEGG pathway enrichment analysis showed that they were mostly enriched in the prolactin signaling pathway, Ras signaling pathway, Hippo signaling pathway, and MAPK signaling pathway.

**Table 3:** The potential microRNAs associated with the hub genes.

	Gene	Predicted microRNAs		Gene	Predicted microRNAs
1	COL1A1	hsa-miR-29c-3p	6	FN1	hsa-miR-613
		hsa-miR-29b-3p			hsa-miR-1271-5p
		hsa-miR-29a-3p			hsa-miR-96-5p
		hsa-miR-4500			hsa-miR-1-3p
		hsa-let-7g-5p			hsa-miR-206
2	COL1A2	hsa-miR-29b-3p	7	MMP9	hsa-miR-942-3p
		hsa-miR-29a-3p			hsa-miR-6734-3p
		hsa-miR-29c-3p			hsa-miR-3713
		hsa-miR-4458			hsa-miR-4450
		hsa-let-7d-5p			hsa-miR-6792-3p
3	COL3A1	hsa-miR-29c-3p	8	COL5A1	hsa-miR-29a-3p
		hsa-miR-29b-3p			hsa-miR-29c-3p
		hsa-miR-29a-3p			hsa-miR-29b-3p
		hsa-miR-4458			hsa-miR-493-3p
		hsa-let-7d-5p			hsa-miR-135a-5p
4	COL5A2	hsa-miR-29a-3p	9	COL4A2	hsa-miR-4458
		hsa-miR-29c-3p			hsa-miR-29b-3p
		hsa-miR-29b-3p			hsa-miR-29c-3p
		hsa-miR-4458			hsa-miR-29a-3p
		hsa-let-7d-5p			hsa-miR-98-5p
5	COL4A1	hsa-miR-29b-3p	10	COL6A3	hsa-miR-133a-3p.1
		hsa-miR-29c-3p			hsa-miR-29a-3p
		hsa-miR-29a-3p			hsa-miR-29c-3p
		hsa-miR-124-3p.1			hsa-miR-29b-3p
		hsa-miR-140-3p.1			hsa-miR-148a-3p

## Discussion

The study of a cancer’s molecular mechanism guides its classification, treatment, and the progress of its targeted immunotherapy. Large-scale research and clinical trials have provided individualized GC treatment possibilities. Despite substantial progress in understanding the underlying molecular mechanism and implementing new therapeutic strategies, the five-year survival rate remains low. The tumorigenesis mechanism remains poorly understood. Therefore, it is crucial to identify novel biomarkers and therapeutic targets involved in GC tumor progression.

Recent studies achieved preliminary results by screening biomarkers of different pathological GC types (Durães et al., 2014; Gravalos & Jimeno, 2008; Rong et al., 2018). In our study, we screened multiple datasets to find more GC biomarker candidates and prove their prognostic value. From three microarray datasets, we identified nine major hub genes: COL1A1, COL1A2, COL3A1, COL5A2, COL4A1, FN1, COL5A1, COL4A2, and COL6A3.

We found that eight of the nine hub genes came from the collagen gene family, which participates in the formation of collagen in extracellular matrix proteins. The collagen family consists of 28 members numbered with Roman numerals (Ricard-Blum, 2011). Previous studies have found that abnormal collagen gene expression is usually related to connective tissue disease or osteoporosis (Wu, Yu & Zhou, 2017; Yamaji, 2017). Type I collagen is the most abundant protein in connective tissue and its increased expression is closely related to fibrotic lesions (Yamaji, 2017). Recent evidence has shown that COL1A1 and COL1A2’s mRNA and protein levels are commonly overexpressed in GC patients (Li, Ding & Li, 2016). Moreover, high COL1A1 and COL1A2 expression may predict poor clinical outcomes for GC patients (Rong et al., 2018). COL3A1 overexpression has been confirmed in multiple cancers, such as bladder cancer, while the impact of COL3A1 expression level in GC is not completely understood (Gao et al., 2016; Liu et al., 2018; Yuan et al., 2017). Lower COL5A2 expression indicates better overall survival in bladder cancer patients, suggesting that it is a prognostic biomarker (Li et al., 2017; Zeng et al., 2018). Although bioinformatics analysis has suggested that COL5A2 is a candidate GC biomarker, its precise regulatory mechanism is still unclear (Wang, 2017). Previous studies suggested that COL4A1 was upregulated in multiple malignancies including GC, and that elevated COL4A1 expression might confer trastuzumab resistance in GC patients (Huang et al., 2018; Miyake et al., 2017). COL4A1’s expression level and mechanism requires further study. There have also been few reports on FN1, COL5A1, COL4A2, and COL6A3 genes. Previous studies on COL1A1, COL1A2, COL4A1, and COL5A2 have confirmed the expression dysregulation of these biomarkers (Huang et al., 2018; Li, Ding & Li, 2016; Zeng et al., 2018). In our study, we identified potential new biomarkers COL3A1, COL5A1, COL4A2, and COL6A3. Prior research on collagen genes mainly focused on connective tissue disease, muscle and ligament-related diseases, or the association between hub genes and tumors (Huang et al., 2018; Li, Ding & Li, 2016; Yuan et al., 2017; Zeng et al., 2018). More attention should be given to the collagen gene family’s prognostic and therapeutic value in tumorigenesis, particularly in GC.

The collagen gene family may play a role in the proliferation, invasion, and metastasis of GC cells. Enrichment analysis and miRNA prediction results provide the potential mechanisms for the collagen gene family’s involvement in GC development (Figs. 2 and 10, Table 3). Previous studies have confirmed these mechanisms (Ao et al., 2018). Silencing COL1A2 and COL6A3 can inhibit the proliferation, migration, and invasion of GC cells, and can promote cell apoptosis through the PI3k-Akt signaling pathway (Ao et al., 2018). MiR-129-5p and let-7i miRNA are reported to participate in GC development via COL1A1 expression (Shi et al., 2019; Wang & Yu, 2018). The key genes’ involvement in GC is currently poorly understood, but our enrichment analysis results provided potential pathways that can be validated by further experiments.

It is worth noting that intratumor stroma proportions have been proposed as significant indicators of GC prognosis (Lee et al., 2017). Previous studies have suggested that a high matrix proportion in GC patients means a poor prognosis (Ham, Lee & Hur, 2019; Kemi, Eskuri & Kauppila, 2019; Lee et al., 2017). Collagen genes play a crucial role in cell matrix formation, and their abnormal expression may lead to changes in matrix proportions. Our study found that a high key collagen gene expression indicated a poor GC prognosis. This partly confirms the above view from a bioinformatics perspective. However, the regulatory mechanism of key collagen genes in GC remains unclear. The identified enrichment pathway and microRNAs may help clarify the mechanism, and can be further applied in clinical prognosis evaluation and treatment.

The current study is only a preliminary report. We cannot ignore the possibility of heterogeneous results in integrated bioinformatics analysis due to sample source and quantity limitations. Although the nine hub genes showed remarkable clinical potential in our survival analysis, further basic and clinical trial studies are needed.

## Conclusion

In summary, we identified DEGs that may be involved in GC initiation or progression. We identified a total of 159 DEGs and nine hub genes as potential prognostic GC biomarkers, and validated them using preliminary survival analysis. Our study provides new therapeutic targets for GC treatment and suggests data mining and integration as promising tools in malignant tumor biomarker detection. Since tumor biomarkers need to be validated by clinical data, further experiments should be conducted to confirm our conclusions.

## Additional Information and Declarations

### Data Availability

The following information was supplied regarding data availability: Data is available at NCBI GEO: GSE54129, GSE65801, GSE79973.

## References

[ref-1] Ao R, Guan L, Wang Y, Wang JN (2018). Silencing of COL1A2, COL6A3, and THBS2 inhibits gastric cancer cell proliferation, migration, and invasion while promoting apoptosis through the PI3k-Akt signaling pathway. Journal of Cellular Biochemistry.

[ref-2] Bader GD, Hogue CW (2003). An automated method for finding molecular complexes in large protein interaction networks. BMC Bioinformatics.

[ref-3] Barrett T, Wilhite SE, Ledoux P, Evangelista C, Kim IF, Tomashevsky M, Marshall KA, Phillippy KH, Sherman PM, Holko M, Yefanov A, Lee H, Zhang N, Robertson CL, Serova N, Davis S, Soboleva A (2013). NCBI GEO: archive for functional genomics data sets–update. Nucleic Acids Research.

[ref-4] Bray F, Ferlay J, Soerjomataram I, Siegel RL, Torre LA, Jemal A (2018). Global cancer statistics 2018: GLOBOCAN estimates of incidence and mortality worldwide for 36 cancers in 185 countries. CA: A Cancer Journal for Clinicians.

[ref-5] Cerami E, Gao J, Dogrusoz U, Gross BE, Sumer SO, Aksoy BA, Jacobsen A, Byrne CJ, Heuer ML, Larsson E, Antipin Y, Reva B, Goldberg AP, Sander C, Schultz N (2012). The cBio cancer genomics portal: an open platform for exploring multidimensional cancer genomics data. Cancer Discovery.

[ref-6] Chen X, Leung SY, Yuen ST, Chu KM, Ji J, Li R, Chan AS, Law S, Troyanskaya OG, Wong J, So S, Botstein D, Brown PO (2003). Variation in gene expression patterns in human gastric cancers. Molecular biology of the cell.

[ref-7] Cho JY, Lim JY, Cheong JH, Park YY, Yoon SL, Kim SM, Kim SB, Kim H, Hong SW, Park YN, Noh SH, Park ES, Chu IS, Hong WK, Ajani JA, Lee JS (2011). Gene expression signature-based prognostic risk score in gastric cancer. Clinical cancer research.

[ref-8] Cui J, Chen Y, Chou WC, Sun L, Chen L, Suo J, Ni Z, Zhang M, Kong X, Hoffman LL, Kang J, Su Y, Olman V, Johnson D, Tench DW, Amster IJ, Orlando R, Puett D, Li F, Xu Y (2011). An integrated transcriptomic and computational analysis for biomarker identification in gastric cancer. Nucleic acids research.

[ref-9] DÉrrico M, de Rinaldis E, Blasi MF, Viti V, Falchetti M, Calcagnile A, Sera F, Saieva C, Ottini L, Palli D, Palombo F, Giuliani A (2009). Genome–wide expression profile of sporadic gastric cancers with microsatellite instability. European journal of cancer.

[ref-10] Durães C, Almeida GM, Seruca R, Oliveira C, Carneiro F (2014). Biomarkers for gastric cancer: prognostic, predictive or targets of therapy. Virchows Archiv : an International Journal of Pathology.

[ref-11] Ferreira P, Oliveira MJ, Beraldi E, Mateus AR, Nakajima T, Gleave M, Yokota J, Carneiro F, Huntsman D, Seruca R, Suriano G (2005). Loss of functional E-cadherin renders cells more resistant to the apoptotic agent taxol in vitro. Experimental Cell Research.

[ref-12] Fife BT, Pauken KE (2011). The role of the PD-1 pathway in autoimmunity and peripheral tolerance. Annals of the New York Academy of Sciences.

[ref-13] Gao YF, Mao XY, Zhu T, Mao CX, Liu ZX, Wang ZB, Li L, Li X, Yin JY, Zhang W, Zhou HH, Liu ZQ (2016). COL3A1 and SNAP91: novel glioblastoma markers with diagnostic and prognostic value. Oncotarget.

[ref-14] Gomez-Martín C, Lopez-Rios F, Aparicio J, Barriuso J, García-Carbonero R, Pazo R, Rivera F, Salgado M, Salud A, Vázquez-Sequeiros E, Lordick F (2014). A critical review of HER2-positive gastric cancer evaluation and treatment: from trastuzumab, and beyond. Cancer Letters.

[ref-15] Gravalos C, Jimeno A (2008). HER2 in gastric cancer: a new prognostic factor and a novel therapeutic target. Annals of Oncology.

[ref-16] Ham IH, Lee D, Hur H (2019). Role of cancer-associated fibroblast in gastric cancer progression and resistance to treatments. Journal of Oncology.

[ref-17] He J, Jin Y, Chen Y, Yao HB, Xia YJ, Ma YY, Wang W, Shao QS (2016). Downregulation of ALDOB is associated with poor prognosis of patients with gastric cancer. OncoTargets and Therapy.

[ref-18] Huang R, Gu W, Sun B, Gao L (2018). Identification of COL4A1 as a potential gene conferring trastuzumab resistance in gastric cancer based on bioinformatics analysis. Molecular Medicine Reports.

[ref-19] Huang DW, Sherman BT, Tan Q, Collins JR, Alvord WG, Roayaei J, Stephens R, Baseler MW, Lane HC, Lempicki RA (2007). The DAVID Gene Functional Classification Tool: a novel biological module-centric algorithm to functionally analyze large gene lists. Genome Biology.

[ref-20] Kanehisa M (2002). The KEGG database. Novartis Foundation Symposium.

[ref-21] Kemi N, Eskuri M, Kauppila JH (2019). Tumour-stroma ratio and 5-year mortality in gastric adenocarcinoma: a systematic review and meta-analysis. Scientific Reports.

[ref-22] Lee D, Ham IH, Son SY, Han SU, Kim YB, Hur H (2017). Intratumor stromal proportion predicts aggressive phenotype of gastric signet ring cell carcinomas. Gastric Cancer.

[ref-23] Li J, Ding Y, Li A (2016). Identification of COL1A1 and COL1A2 as candidate prognostic factors in gastric cancer. World Journal of Surgical Oncology.

[ref-24] Li S, Liu X, Liu T, Meng X, Yin X, Fang C, Huang D, Cao Y, Weng H, Zeng X, Wang X (2017). Identification of biomarkers correlated with the TNM staging and overall survival of patients with bladder cancer. Frontiers in Physiology.

[ref-25] Li H, Yu B, Li J, Su L, Yan M, Zhang J, Li C, Zhu Z, Liu B (2015). Characterization of differentially expressed genes involved in pathways associated with gastric cancer. PLOS ONE.

[ref-26] Liu X, Wu J, Zhang D, Bing Z, Tian J, Ni M, Zhang X, Meng Z, Liu S (2018). Identification of potential key genes associated with the pathogenesis and prognosis of gastric cancer based on integrated bioinformatics analysis. Frontiers in Genetics.

[ref-27] Miyake M, Hori S, Morizawa Y, Tatsumi Y, Toritsuka M, Ohnishi S, Shimada K, Furuya H, Khadka VS, Deng Y, Ohnishi K, Iida K, Gotoh D, Nakai Y, Inoue T, Anai S, Torimoto K, Aoki K, Tanaka N, Konishi N, Fujimoto K (2017). Collagen type IV alpha 1 (COL4A1) and collagen type XIII alpha 1 (COL13A1) produced in cancer cells promote tumor budding at the invasion front in human urothelial carcinoma of the bladder. Oncotarget.

[ref-28] Okines AF, Thompson LC, Cunningham D, Wotherspoon A, Reis-Filho JS, Langley RE, Waddell TS, Noor D, Eltahir Z, Wong R, Stenning S (2013). Effect of HER2 on prognosis and benefit from peri-operative chemotherapy in early oesophago-gastric adenocarcinoma in the MAGIC trial. Annals of Oncology.

[ref-29] Raufi AG, Klempner SJ (2015). Immunotherapy for advanced gastric and esophageal cancer: preclinical rationale and ongoing clinical investigations. Journal of Gastrointestinal Oncology.

[ref-30] Ricard-Blum S (2011). The collagen family. Cold Spring Harbor Perspectives in Biology.

[ref-31] Rong L, Huang W, Tian S, Chi X, Zhao P, Liu F (2018). COL1A2 is a novel biomarker to improve clinical prediction in human gastric cancer: integrating bioinformatics and meta-analysis. Pathology Oncology Research.

[ref-32] Shi Y, Duan Z, Zhang X, Zhang X, Wang G, Li F (2019). Down-regulation of the let-7i facilitates gastric cancer invasion and metastasis by targeting COL1A1. Protein & Cell.

[ref-33] Siegel RL, Miller KD, Jemal A (2015). Cancer statistics, 2015. CA: a Cancer Journal for Clinicians.

[ref-34] Szklarczyk D, Franceschini A, Wyder S, Forslund K, Heller D, Huerta-Cepas J, Simonovic M, Roth A, Santos A, Tsafou KP, Kuhn M, Bork P, Jensen LJ, von Mering C (2015). STRING v10: protein-protein interaction networks, integrated over the tree of life. Nucleic Acids Research.

[ref-35] Wang Y (2017). Transcriptional regulatory network analysis for gastric cancer based on mrna microarray. Pathology Oncology Research.

[ref-36] Wang Q, Wen YG, Li DP, Xia J, Zhou CZ, Yan DW, Tang HM, Peng ZH (2012). Upregulated INHBA expression is associated with poor survival in gastric cancer. Medical oncology.

[ref-37] Wang Q, Yu J (2018). MiR-129-5p suppresses gastric cancer cell invasion and proliferation by inhibiting COL1A1. Biochemistry and Cell Biology = Biochimie et Biologie Cellulaire.

[ref-38] Wu J, Yu M, Zhou Y (2017). Association of collagen type I alpha 1 +1245G/T polymorphism and osteoporosis risk in post-menopausal women: a meta-analysis. International Journal of Rheumatic Diseases.

[ref-39] Yamaji K (2017). Immunoadsorption for collagen and rheumatic diseases. Transfusion and Apheresis Science.

[ref-40] Yuan L, Shu B, Chen L, Qian K, Wang Y, Qian G, Zhu Y, Cao X, Xie C, Xiao Y, Wang X (2017). Overexpression of COL3A1 confers a poor prognosis in human bladder cancer identified by co-expression analysis. Oncotarget.

[ref-41] Zeng XT, Liu XP, Liu TZ, Wang XH (2018). The clinical significance of COL5A2 in patients with bladder cancer: a retrospective analysis of bladder cancer gene expression data. Medicine.

